# Does Kin Recognition and Sib-Mating Avoidance Limit the Risk of Genetic Incompatibility in a Parasitic Wasp?

**DOI:** 10.1371/journal.pone.0013505

**Published:** 2010-10-19

**Authors:** Marie Metzger, Carlos Bernstein, Thomas S. Hoffmeister, Emmanuel Desouhant

**Affiliations:** 1 Université de Lyon; Université Lyon 1; CNRS; UMR 5558, Laboratoire de Biométrie et Biologie Evolutive, Villeurbanne, France; 2 Institut für Oekologie, Universitaet Bremen, Fachbereich 2, Bremen, Germany; Royal Holloway University of London, United Kingdom

## Abstract

**Background:**

When some combinations of maternal and paternal alleles have a detrimental effect on offspring fitness, females should be able to choose mates on the basis of their genetic compatibility. In numerous Hymenoptera, the sex of an individual depends of the allelic combination at a specific locus (single-locus Complementary Sex Determination), and in most of these species individuals that are homozygous at this sexual locus develop into diploid males with zero fitness.

**Methods and Findings:**

In this paper, we tested the hypothesis of genetic incompatibility avoidance by investigating sib-mating avoidance in the solitary wasp parasitoid, *Venturia canescens*. In the context of mate choice we show, for the first time in a non-social hymenopteran species, that females can avoid mating with their brothers through kin recognition. In “no-choice” tests, the probability a female will mate with an unrelated male is twice as high as the chance of her mating with her brothers. In contrast, in choice tests in small test arenas, no kin discrimination effect was observed. Further experiments with male extracts demonstrate that chemical cues emanating from related males influence the acceptance rate of unrelated males.

**Conclusions:**

Our results are compatible with the genetic incompatibility hypothesis. They suggest that the female wasps recognize sibs on the basis of a chemical signature carried or emitted by males possibly using a “self-referent phenotype matching” mechanism.

## Introduction

Sexual selection theory predicts that females, being the sex that generally invest more in offspring, would be selective when choosing a male [1], [2]. Female preferences have been selected for mates providing the most material benefits (direct selection, reviewed by [3]) or genetic benefits (indirect selection, reviewed by [4]). In the latter case, preferred males carry genes which confer by themselves a higher fitness, improving viability or attractiveness of offspring (discussed and reviewed by [5]–[7]). Whether the main genetic benefits are “good genes”, “compatible genes” or “diverse genes” is still debated [8]. Females may prefer males with genes compatible with their own genotypes, rather than males with “good genes” [2], [9], [10]. In turn, they should avoid males with “incompatible genes”, i.e. reject matings leading to allelic combinations with a deleterious effect on offspring viability or fertility (genetic incompatibility hypothesis [11], [12]). Incest avoidance can be regarded as a special case of genetic incompatibility avoidance [9], [13]. Indeed, while inbreeding depression comes to some extent from an increase in the phenotypic expression of deleterious recessive alleles, matings between relatives increase the risk of homozygosity in offspring [14], [15].

In the Hymenoptera, a group known for its ecological and economic importance [16], the haplodiploid genetic system is traditionally expected to prevent detrimental effects of low genetic diversity such as inbreeding depression, because the genetic load can be purged via haploid males [17]. However, sex in many of the species depends on the allelic combination at a specific locus [18]–[21]: the single-locus complementary sex determination, henceforth referred to as sl-CSD which may lead to inbreeding costs. Sl-CSD offers an excellent opportunity to test the genetic incompatibility hypothesis by investigating mate choice for complementary genotypes. In these haplodiploid organisms, the sl-CSD locus determines gender, with heterozygous individuals developing as females and haploids and homozygous diploids developing as males [18]–[20]. Genetic incompatibility occurs in diploid eggs homozygous at the sl-CSD locus which give rise to diploid male phenotypes that are sterile in most of the species studied so far, given they are viable at all (but see [22]). Hence, matings resulting in an increased risk of homozygosity at the CSD locus, such as matings between relatives, potentially have a high cost and should be avoided. A female that mates with a male carrying the same sex allele will waste half of her fertilized eggs because these will develop into diploid males (matched mating [19]). When mating takes place between siblings (sibs), the risk of matched mating, i.e., the alleles of the two partners match, is 50%. An inter-sexual conflict could appear since, under the hypothesis that males are not effectively sperm limited, they should mate regardless of their relatedness with the females.

In this paper, we test the genetic incompatibility hypothesis by investigating whether genetic compatibility occurs during mate choice in a hymenopteran parasitoid wasp, *Venturia canescens* (Gravenhorst) with sl-CSD [23]. We conducted a set of three experiments to investigate sib-mating avoidance and the associated behavioural mechanism of kin recognition in this species. In the first one, to test whether matings occur with a higher probability with non-relatives than with relatives, females were either exposed to two sib males or two non-sib males. We predicted that matings with non-relatives should occur with higher probability and should occur faster after the encounter of the sexes. In a second experiment, females were offered a choice between a sib and a non-sib male; we expected that females should be able to avoid sib matings and should prefer to mate with non-sib males. Finally a third experiment aimed at investigating the mechanism of sib recognition. Here, we exposed females to the odour of sib-males in the presence of non-sib males. Given sib-mating avoidance is based on volatile chemical signatures, we expected that female mating propensity with non-sib males would decrease in the presence of sib-odour.

## Methods

### Biological model

Under natural conditions, arrhenotokous *Venturia canescens* females (i.e, with haplodiploid sex determination) parasitize pyralid moth larvae developing in dried fruits such as figs, carobs, almonds, dates or loquats [24]. This parasitoid is a solitary species and thus only a single offspring completes its larval development and emerges from each host irrespective of the number of eggs laid in it. Virgin *V. canescens* females emit chemicals that in combination with host kairomones attract males. In turn, males do not attract virgin females at a distance [25]. Our knowledge of how mating partners encounter each other under field conditions is largely incomplete as a consequence of the small size of the species that renders observations difficult. Courtship behaviours have been described in van Santen & Schneider [26] and females do not choose the male according to its size [27]. Like 80% of the studied parasitoid wasp species [28], *V. canescens* females are monandrous and thus only mate once in a lifetime [25]. Conversely, males can mate more than once (E. Desouhant pers. obs.). Therefore mate choice in general, and female avoidance of a sib in particular, should have a great adaptive impact in this species because of the CSD.

### Rearing facilities

Wasps were reared on *Ephestia kuehniella* (Lepidoptera: Pyralidae) larvae maintained in wheat semolina medium. They were fed with 50% water-diluted honey. Insect cultures (culture boxes) were kept under constant laboratory conditions (24±1°C, 70±10% Hr, DL 12∶12). To ensure genetic diversity in the wasp cultures, strains used in the first two experiments had been established from large scale field sampling: parasitized hosts were collected from eight sites (P1-P4 and P6-P9 in [29]) along a 20 km transect near Valence, south-eastern France, on 17^th^ July, 2^nd^ and 23^rd^ August 2005. Culture boxes contained a mixture of females from the different sampling localities. Experiments 1 and 2 were conducted between January and March 2006. The third experiment was carried out in July 2008, with wasps from Nice, south-eastern France. The culture was started also with a large number of females (more than 50, E. Desouhant Com. Pers.) sampled during three days (19, 25 and 28^th^ August) in 2005.

### Parasitoid families

In order to obtain individuals with different relatedness, families of wasps were formed. For experiments 1 and 2 (see below), virgin females from different culture boxes were individually isolated during 24 h in plastic tube (70×30 mm) immediately after emergence. They were allowed to mate once with a male among three non-sib males from another culture box to reduce the risks of matched mating. For experiment 3, we slightly changed the procedure to obtain mated females. Ten to 20 freshly emerged females from different culture boxes were gathered in a plastic container with males of various ages. The sex ratio in the container was approximately 1 female for 6 males. A large majority of females accept mating within the first ten minutes of contact with males (see results of Experiment 1). Even if some females have mated more than once [25], brothers are always similarly related to their sisters as a consequence of haplodiploidy.

For all experiments, each female was isolated the day after mating and allowed to oviposit on a patch of 30 third-instar host larvae for 2 h. Host patches were set up, 7 days before parasitisation, in plastic boxes (80×50 mm) containing 15 g of semolina. To increase sample size of brothers and sisters available for experiments, five days after parasitisation, females were provided with a new host patch under the same conditions. A few days before offspring emergence, potentially parasitized host pupae were placed individually in gelatine capsules (Ø 7.95×23 mm). The capsules were checked daily and each newly emerged wasp was transferred to a plastic tube (70×30 mm) and its family was recorded. This procedure assures that brothers and sisters were separated before emergence and maintained without contact with conspecifics until the experiments started. All the females and males in the tubes were provided with a drop of 50% water –diluted honey until 2 h before the tests.

### Experiment 1: Mating motivation in presence of either sibs or non sibs

Our aim was to investigate whether relatedness between mating partners influenced frequency and latency of mate acceptance.

Each female tested was given the opportunity to choose a mate among either two of her brothers or two unrelated males (*Relatedness* treatment). The two males unrelated to the female were brothers. The females were one day old. We offered two males per female (1) to allow mate choice and to increase the proportion of females accepting a male for mating [26] and (2) to be able to compare results of experiments 1 and 2. Occurrence and time spent before mating for each female were recorded during 30 min. The two males were introduced together to the plastic tube (70×30 mm) containing the female. The males were one or two-days-old (mean 1.5) and each experimental day the same number of males of each age were randomly assigned to the 2 experimental *Relatedness* treatments. There was no effect of male age on mating propensity (χ^2^ = 1.49, df = 2, p = 0.48). Thirty-four females were tested for each experimental combination during 11 days. Only one female, randomly chosen, from each of the 200 families formed was used in this experiment to avoid pseudo-replication via a brood effect. In others words, the 68 females used were all taken from different ‘families’.

### Experiment 2: Mate choice between sib and non-sib

This experiment aimed at testing whether females avoid sib-mating when they have the choice between a brother and an unrelated male. This experiment was conducted on the same dates as Experiment 1, with wasps of the same broods, under similar experimental conditions, and recording the same variables. To distinguish whether a female mated with her brother or the unrelated male, we pierced the wing base of one male with a fine insect pin (hole diameter <0.5 mm on a wing measuring 15 mm) at least 20 h before the beginning of the observation period. The male with the pierced wing was randomly drawn between the two males. After the end of the observation, the male that copulated was identified by examining its wings using a binocular microscope. All males and females used were one day old and 88 females were tested. Only one female per family was tested.

### Experiment 3: Mechanism of sib recognition

The first two experiments produced apparently contradictory results (see below) which could be due to the presence of sib volatiles hindering the recognition of sib and non sib males in Experiment 2. Females may not be able to distinguish between sib and non-sib males in close proximity, if the cue of kin recognition is a volatile chemical compound present throughout the entire experimental vial. To test this hypothesis, we replaced the sib-male that was present in experiment 2, with the chemical extract from a sib male (treatment 1), a non-sib male (treatment 2) or pure solvent (control treatment). Under our hypothesis, we expected that the probability of mate acceptance with the non-sib male would be higher in presence of non-sib extract than with sib extract. We also expected that mating probabilities were similar between treatment 2 and control.

The chemical extracts from a sib male and a non-sib male were prepared from males frozen at −20°C on the day of their emergence (conserved less than 15 days). To make a male extract, its entire body was crushed in 500 µl of pentane. The mixture was centrifuged for 30 min at 3000 rpm; the supernatant liquid was recovered and concentrated to approximately 100 µl using a vacuum concentrator. The extracts were used less than 4 hours after being prepared.

One-day-old virgin females of the different families were placed with a non-sib male after being exposed for 15 min to the crude extract from a sib male, a non-sib male or to pure solvent. According to the experimental treatment, 40 µl of male extract or pure solvent were deposited on a filter paper (1 cm^2^), which was placed, after few seconds of solvent evaporation, together with the test female in an Eppendorf micro test tube (1,5 ml). After 15 min of exposure, giving the female ample time to perceive the male extract, the tested female was transferred to a plastic tube (as in experiments 1 and 2) with the remaining volume of extract (60 µl) deposited on the filter paper. A non-sib male was immediately introduced into the tube and the occurrence of mating was recorded during a period of 30 min. Thirty two replicates per experimental treatment were conducted (leading to 96 females tested within 4 days). Females tested had been randomly taken from 43 families (mean ± SD of 2.2±1.4 females used per family) and randomly assigned to the three treatments.

### Data analysis

The influence of the *Relatedness* with males (sib or non sib) on the proportions of matings in Experiment 1 and 2 was analysed with chi-square tests. In experiment 1, the effect of *Relatedness* on the probability of mate acceptance within the 30 min observation period (i.e. the latency to mate) was assessed using a non-parametric survival analysis (Log-rank test) allowing for right censored data (i.e. when no mating was observed during the observation period). Each observed mating was considered as an event and the mating latency was the survival time.

Occurrences of matings in Experiment 3 were analyzed using a mixed model (with binomial error and logit link function), with *Treatment* (3 treatment levels) as an explanatory variable (fixed effect) and family as a random effect to allow for the fact that more than one female per family was tested in this experiment. Since statistical treatment contrasts of interest were “non sib” *vs. “*sib” and “non sib” *vs.* “control”, we present only results of Wald-tests on the coefficients estimating the effect of the *Treatment* factor. The variance of the random effect was insignificant (<10^−6^) and fixed effect estimates and SE did not change (R library *lme4*).

All data analyses were performed with statistical procedures in R [30].

## Results

### Experiment 1: Mating motivation in presence of either sibs or non sibs

Females accepted a mate with higher probability in the presence of non-sibs. Seventy nine percent of females (27/34) mated when they were in presence of non sib males. Only 41% (14/34) mated when they were with sibs. Virgin females were significantly more likely to mate with non-sib than sib males (*Relatedness* effect: χ^2^ = 8.84, df = 1, p = 0.003).

The probability of mating within the 30 minutes of observation (i.e. the latency to mate) was significantly influenced by *Relatedness*. It was higher in presence of non-sib than in presence of sib males (log-rank test: χ^2^ = 8.1, *df* = 1, *p* = 0.004). Most of the observed matings occurred during the first 10 minutes of observation (median (quartiles 25, 75%): 335 s (88, 882)).

### Experiment 2: Mate choice between a sib and a non-sib

**Figure 1:**
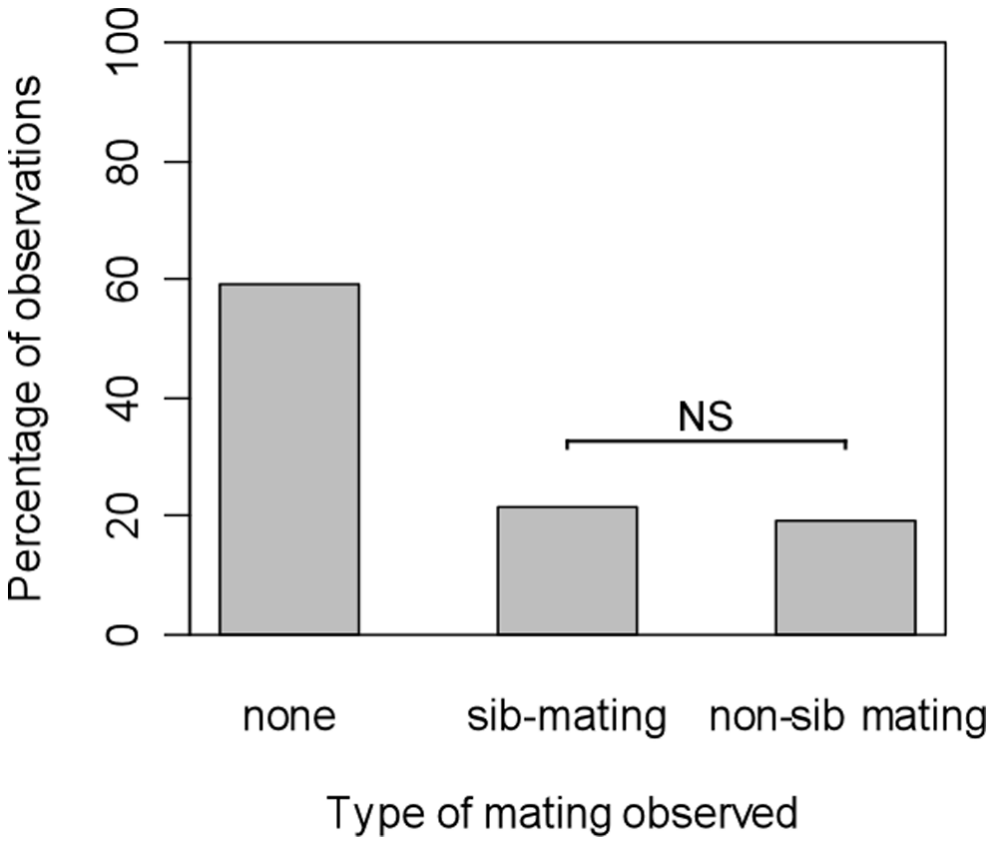
Percentage of observation trials in which there was either no mating, a mating between sibs, or a mating between non-sibs (experiment 2). Each female had the choice between a brother and an unrelated male. NS: non significant; * : p<0.05.

Among the 88 females offered a brother and an unrelated male, 52 did not mate. Thirty six accepted a mate during the observation period (40.9%). The proportion of females mating with a sib (19/36) was not significantly different from that of females mating with a non-sib male (17/36, χ^2^ = 0.11, *df* = 1, *p* = 0.74, Figure 1). Female choice was not influenced by the hole in the wing used to distinguish males (Fisher exact test, *p* = 0.736). The proportion of mate acceptance was not significantly different from the proportion observed in the treatment with one-day-old females exposed to brothers in the experiment 1 (χ^2^ = 0.0318, *df* = 1, *p* = 0.85).

### Experiment 3: Mechanism of sib recognition

**Figure 2:**
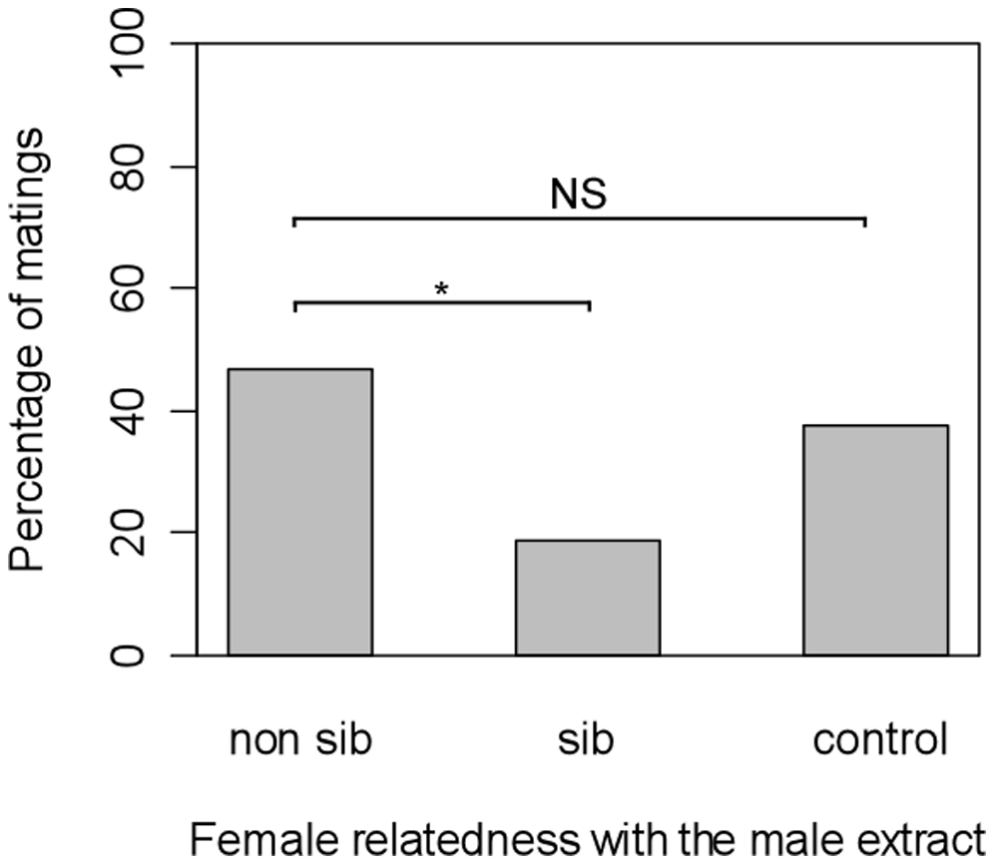
Percentage of females accepting to mate with a non-sib male according to their relatedness with the male extract (N = 32/treatment; experiment 3). * : p<0.05.

Matings with the non-sib male were less frequent when the pair perceived the chemical extract of a sib male than in the presence of a non-sib male extract (*Treatment* factor: χ^2^ = 6.09, *df* = 2, *p* = 0.048, “non sib” *vs.* “sib”: *Z* = 2.332, *p* = 0.0197, Figure 2). Being exposed to an extract of non-sib males had no effect on the proportion of females accepting to mate with the non sib male (“control” *vs.* “non sib” extracts, *Z* = 0.758, *p* = 0.44).

## Discussion

Our study shows for the first time that females of a solitary parasitoid wasp species respond differentially to genetically related conspecifics for mate choice. They avoid mating with their brothers and prefer unrelated males as expected under the “genetic incompatibility hypothesis”. The avoidance of sib-mating limits the risks of matched mating for sl-CSD and therefore the costs of genetic inbreeding. Our results also strongly suggest that the mechanism of kin recognition relies on chemical compounds carried or released by males.

The probability of mating in the arrhenotokous *V. canescens* females is twice as high in the presence of two unrelated males as in the presence of two of her brothers (experiment 1). This indicates that this species exhibits kin recognition during mating choice. Kin discrimination ability has been described in the context of superparasitism (i.e. several broods in the same host) for thelytokous *V. canescens* (i.e. females that do not mate and produce only females from diploid unfertilized eggs). In these females the probability of laying an egg in an already parasitized host depends of the relatedness of the female with the progeny within the host [31]. Kin recognition was also described in another non-social and solitary wasps in a context of nest defence [32], [33] *V. canescens* can be added to the few insect species for which incest avoidance through kin discrimination during mate choice has been reported: the cricket *Gryllus bimaculatus* [34], the cockroach *Blattella germanica* [35], and social insects such as bees [36], the ants *Iridomyrmex humilis and Plagiolepis pygmaea* [37], [38], and the termite *Zootemopsis nevadensis* [39]. In the bumblebee *Bombus terrestris* (with CSD), mating behaviour is also affected by relatedness of partners [40].

Incest avoidance in *V. canescens* is consistent with the model of genetic complementarity, which assumes that females do not always choose a male with intrinsically superior genes. They may instead choose males with whom they have higher genetic compatibility, i.e. the viability of offspring depends on the interaction between the male and female genotypes [13]. Many studies of genetic complementarity have focused on polyandrous species where the potential for postcopulatory female choice exists [41]. However, in monoandrous species one would expect that some precopulatory indications of a male's relatedness are used during mate choice.

Our results suggest the nature of the cue used for kin recognition. In contrast to the results of experiment 1, there is no female preference for non-sib males when females are given the choice between a sib and a non-sib male (experiment 2). Only 40% of these females accepted to mate with unrelated males, which is the similar percentage to that shown by females in the presence of sibs in experiment 1. This apparent discrepancy between the two experiments might be explained by the presence of chemical volatiles from sib-males in the small mating arena preventing the females from distinguishing between the two males in experiment 2. This hypothesis was supported by the results of experiment 3: whilst the proportion of females accepting to mate with a non-sib male does not differ when they are exposed to non-sib male chemical cue or to the control (pure solvent), this proportion decreases in presence of chemical cues from male siblings. This suggests that sib recognition involves chemical compounds carried or released by *V. canescens* males and that the perception of a brother's chemical compounds inhibits a female's receptivity to mate. It is noteworthy that our ability to detect kin discrimination in *V. canescens* was influenced by the experimental design. Often, preference or selectivity tests are performed either as choice or no-choice test, but results from such tests may differ (e.g. [42]). Here, kin discrimination was only visible under no-choice conditions and appears to have been obscured by the effects of chemical cues from sibs in the small arena choice test. The apparent lack of consistency of our results across experiments indicates that further experimentation will be necessary to detect the true nature of kin recognition and discrimination.

In our experiment we used the whole extract of males (experiment 3). This protocol prevents us from distinguishing whether the chemical signature is carried on the surface of males (cuticular hydrocarbons) and/or whether it is released by males during courtship. The use of a chemical label is consistent with the observation that in the superparasitism context the *V. canescens* females use hydrocarbon profiles that are more variable between non relatives than between sibs [31].

Chemical signatures, such as hydrocarbons for recognition [43]–[45], are widespread and reliable labels, especially in insects [46], [47]. In the solitary parasitoid larvae, *Aleochara bilineata* (a non–social coleopteran), sibs are recognised by use of a chemical cue, present on plugs placed by larvae on the host during parasitization [48]. The gregarious parasitoid (i.e. that lay eggs in clusters) *Bracon hebetor* (with sl-CSD) uses the odour of the host in which brothers and sisters develop for brood-mate avoidance. This is a reliable cue to recognise sibs in gregarious parasitoids, as long as no superparasitism occurs [49]. Since only one adult emerges from a host in *V. canescens*, and because hosts are distributed across different fruits that are generally clumped, females cannot use cues related to a common host patch origin to discriminate between sib and non-sib males. Cuticular hydrocarbons that vary with family origin are used both in social context and incest avoidance in urban cockroaches [45].

To be recognized, a label should be compared to a “template” representing kin [46], [50], [51]. The most frequent mechanisms of recognition are: prior association or phenotype matching [52], [53]. The mechanism of prior association assumes that individuals learn the phenotypes of familiar conspecifics during their early development, thus allowing them to discriminate later familiar from non-familiar conspecifics [54]. In our study, the wasps did not encounter any conspecifics before the test; hence we can exclude this mechanism. Phenotype matching assumes that individuals learn their own phenotypes (self referent phenotype matching, [55]–[60]) or those of their familiar kin, thus allowing them later to compare phenotypes of conspecifics to this learned recognition template [61]. This mechanism assumes a positive correlation between phenotypic and genotypic similarities. In our experiments, the parasitoid larvae grew separately and adults were isolated prior to mating, therefore an individual's own phenotype is the only reference that it can use. Consequently, it might be suggested that *V. canescens* uses self-referent phenotype matching to recognize and avoid sib-matings.

Our work relies on the hypothesis that females choose the males they mate with. However, we cannot rule out that the males may also be choosy (male mate choice, for a review in insects see [62]) and able to discriminate kin. This ability could provide an alternative explanation for the discrepancy between the results of the first two experiments. In the first experiment, the males are brothers (and unrelated to the female). If males are capable of kin discrimination, under a kin selection hypothesis, they would be less aggressive toward a brother, and this could reduce the competition for mates. In the second experiment a sib male is in competition with a non sib and it might behave more aggressively preventing a non kin male from mating with the female. Yet, we should still see a difference between the mating propensities of kin and non-kin males, since the former should avoid mating with the females whereas the latter should try to mate. Thus, given the data, female choice and the effect of the presence of kin chemical traces on female mate choice seem to be the better explanation in *V. canescens*.

Such kin recognition and avoidance behaviour is expected to have been selected for in *V. canescens* as in other Hymenoptera species with sl-CSD to avoid the costs of diploid male production that results from a mating between siblings (see Introduction, but see [63]). Avoidance of sib-mating is a mating bias acting indirectly against genetic incompatibility [2], [8], [11]. In *V. canescens* inbred crosses lead to viable diploid males [23]. Matings with diploid males result in no viable diploid female offspring (X Fauvergue Comm. Pers), as is the case in most species with sl-CSD where diploid males produce sperm (but see for an exception [22], [64], [65]). Of course, inbreeding avoidance in this species may also have been selected for the reduction of deleterious recessive gene expression in diploids and inbreeding depression.

Sl-CSD and sib-mating avoidance may also have severe consequences for population dynamics. Using a modelling approach, Zayed & Packer [66] predicted that the genetic load of sl-CSD is high enough to drive panmictic populations into an extinction vortex when they suffer from a size reduction (see also [67], but see [68]). Results from two cage population experiments with a small number of mated foundresses suggest the existence of costs associated with the production of diploid *V. canescens* males. After 5 months (approx. 8 generations), all cage populations went to extinction because of all male-offspring production (I. Amat and C. Bernstein, unpublished data). In the bumblebee *B. terrestris*, the diploid males produced by matched mating suffer reduced fertility [69] and reduces the survivorship of colonies in the field [70]. Such consequences are likely exacerbated in species that mate only once, as in *V. canescens* [25].

To conclude, inbreeding avoidance via kin recognition is generally expected and reported in social species or in animals living in groups [35], [58], [71], [72], with very few reports for non-social species [73]. As such demonstrating sib-mating avoidance in a solitary wasp is a novel result. It suggests that even in non-social species, the potential costs of matched matings may be strong enough to favour mechanisms of sib-mating avoidance in the field. Hymenoptera with sl-CSD represent suitable model systems to study the genetic mechanisms (genetic diversity at the whole organism level or at the CSD locus) underlying sib-mating avoidance, its adaptive value and its consequences at the population level.
